# Hemichorea-Hemiballismus As Presentation of Toxoplasma gondii Encephalitis in an Immunocompromised Host

**DOI:** 10.7759/cureus.100920

**Published:** 2026-01-06

**Authors:** Chu Keat Lim, Xiong Khee Cheong, Juen Kiem Tan, Ummu Afeera Zainulabid, Hui Jan Tan, Najma Kori, Petrick Ramesh Periyasamy

**Affiliations:** 1 Internal Medicine, Universiti Kebangsaan Malaysia Medical Centre, Kuala Lumpur, MYS; 2 Internal Medicine, International Islamic University Malaysia, Pahang, MYS; 3 Neurology, Universiti Kebangsaan Malaysia Medical Centre, Kuala Lumpur, MYS

**Keywords:** cd4, hemichorea-hemiballismus, hiv aids, toxoplasma encephalitis, toxoplasma gondii infection

## Abstract

*Toxoplasma gondii* encephalitis is a common but serious opportunistic infection in people living with human immunodeficiency virus (PLHIV), particularly those with advanced disease and low CD4 counts. Its classical manifestations include fever, altered mental status, seizures and focal neurological signs. Movement disorders are a less common consequence, with hemichorea-hemiballismus (HCHB) representing a rare but striking manifestation. We describe a 36-year-old man with advanced HIV disease who had defaulted on treatment. He presented with bizarre, involuntary movements of his left upper and lower limbs. His serum Toxoplasma IgG was positive, and brain imaging revealed a basal ganglia lesion leading to the presumptive diagnosis of cerebral toxoplasmosis. He was treated with anti-toxoplasmosis and anti-dyskinetic medications, achieving both clinical and radiological resolution after one month of therapy. A high clinical suspicion for Toxoplasma gondii encephalitis should be maintained in patients with advanced HIV disease who present with rare manifestations such as movement disorders. Timely management, guided by serology and brain imaging, is essential to improve treatment outcomes.

## Introduction

Toxoplasma gondii encephalitis is a severe opportunistic cerebral infection caused by reactivation of the intracellular protozoan Toxoplasma gondii. In immunocompromised individuals, latent tissue cysts acquired from consuming undercooked meat, unwashed vegetables, or contact with cat feces can reactivate, leading to necrotising encephalitis. This opportunistic infection occurs most commonly among people living with HIV (PLHIV), with the greatest risk occurring in those with a CD4 count of <50 cells/µL [1]. The classic manifestations of cerebral toxoplasmosis are those of a focal encephalitis, including headache, fever, confusion, seizures, focal motor weakness, and potentially coma. However, movement disorders, while less common, represent a significant and diagnostically crucial clinical presentation. Reported rates of movement disorders in HIV-associated cerebral toxoplasmosis vary from 2% in contemporary antiretroviral therapy (ART)-treated cohorts to as high as 40% in pre-ART populations [2-5]. Hemichorea-hemiballismus (HCHB) in PLHIV is a rare presentation compared to other movement disorders [3,4]. Involvement of subcortical brain structures, particularly the basal ganglia, is often related to opportunistic infections like toxoplasmosis, especially in patients with advanced HIV disease [2]. Despite advancements in ART and the decline in severe opportunistic infections, this case contributes to the essential educational repository for clinicians in recognising HCHB as a neurological manifestation in individuals with AIDS. It also reinforces the need for early initiation of appropriate therapy to improve outcomes.

## Case presentation

A 36-year-old man with a known history of HIV and hepatitis B co-infection presented to the emergency department with an acute neurological syndrome in December 2023. He had been lost to follow-up for two years after initially starting ART. He presented with a two-day history of involuntary movements affecting his left arm and leg. The symptom was accompanied by slurred speech and involuntary grimacing. He noticed the hyperkinetic symptoms completely resolved during sleep. There was no history of diabetes, substance use or family history of movement disorders. On examination, he was alert and oriented. Neurological examination revealed left-sided hemichorea-hemiballismus (HCHB), with left upper limb hypertonia and hyperreflexia but preserved muscle power of 5/5. An urgent ophthalmology review ruled out ocular toxoplasmosis.

**Figure 1:**
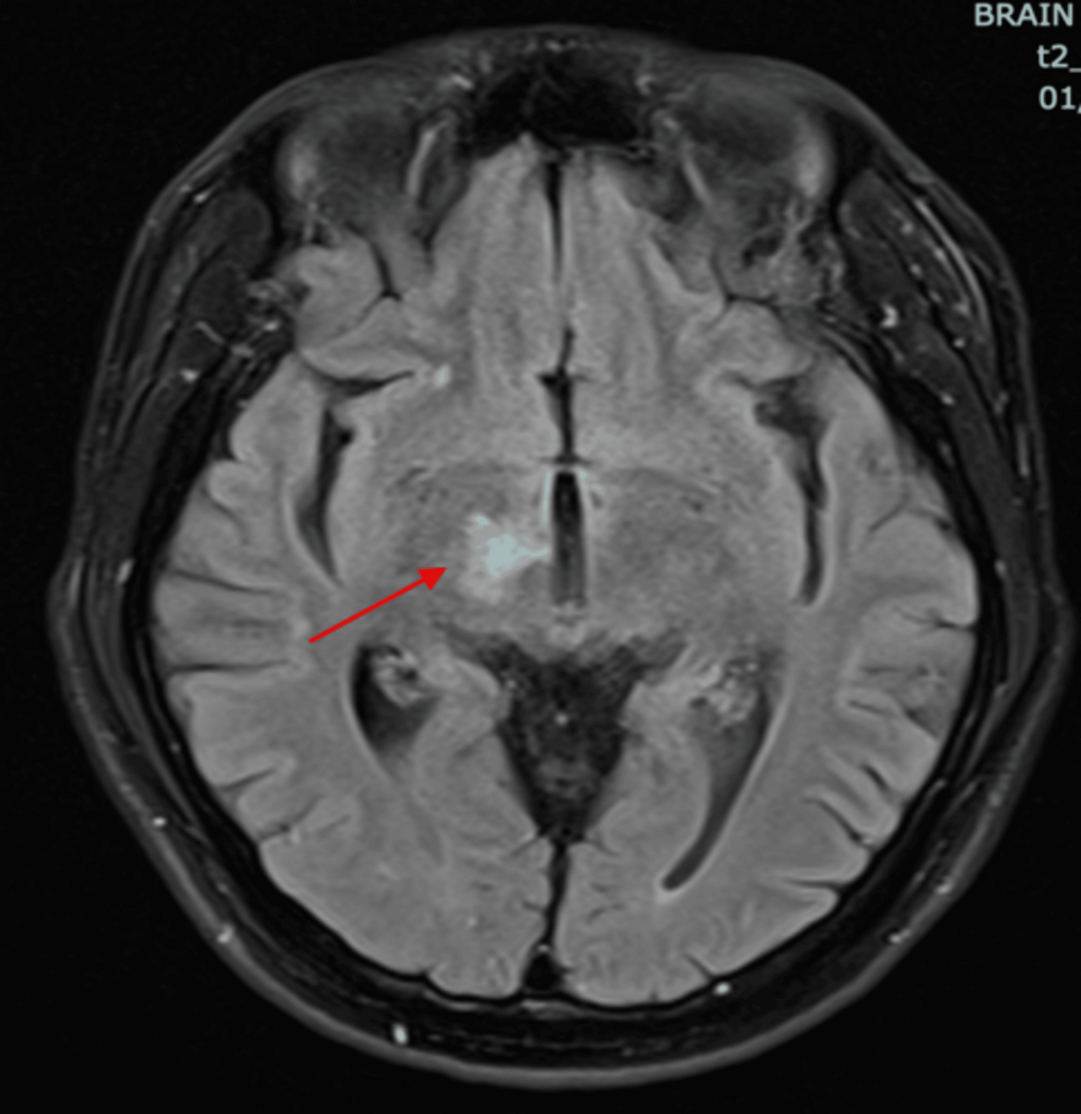
MRI of the brain T2 FLAIR showed hyperintensity at the right thalamus with perilesional edema (thin red arrow). T2-FLAIR: T2-weighted-Fluid-Attenuated Inversion Recovery

**Figure 2:**
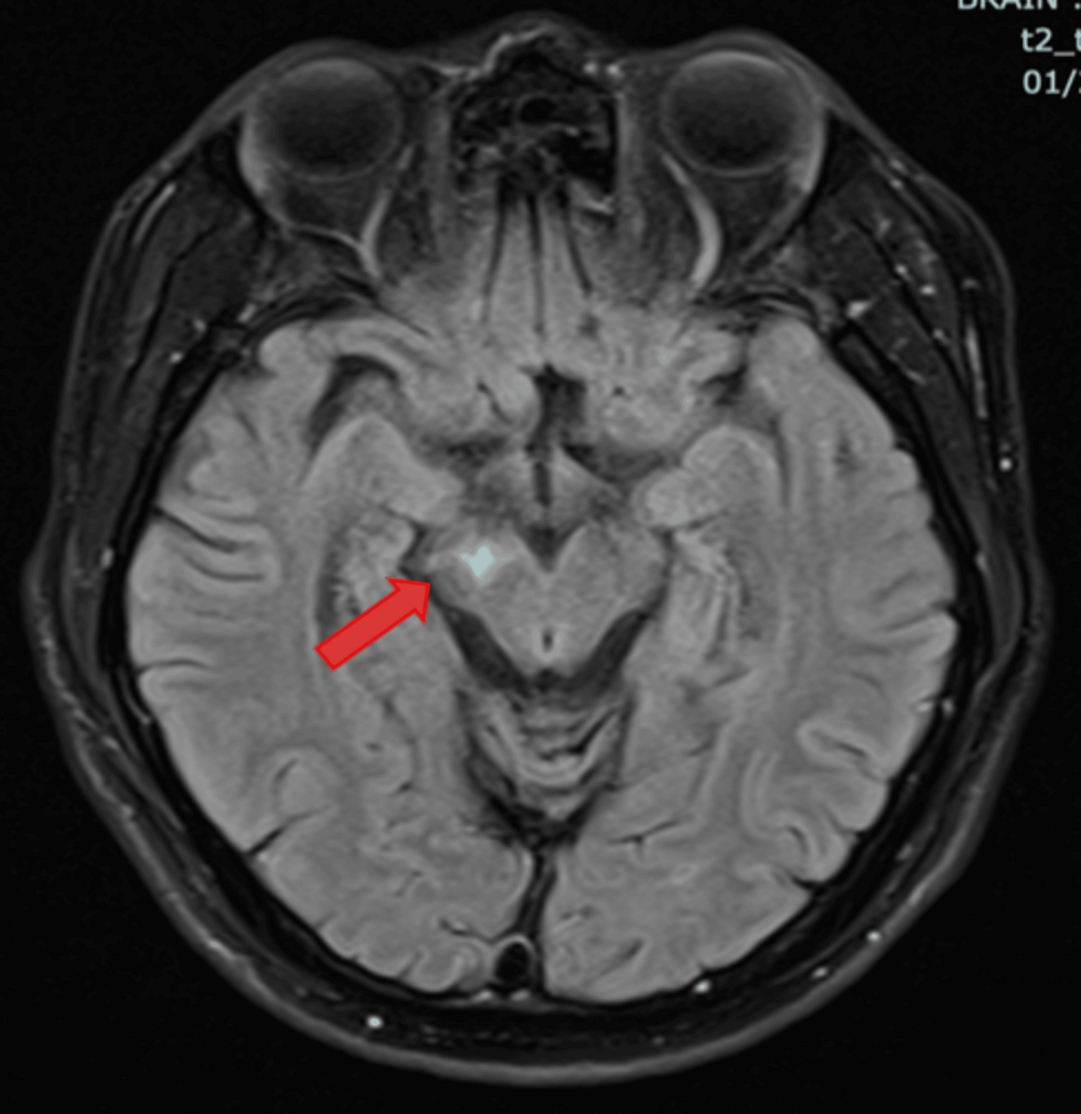
MRI of the brain with T2 FLAIR showed hyperintensity at the right cerebral peduncle (thick red arrow) T2-FLAIR: T2-weighted-Fluid-Attenuated Inversion Recovery

Initial blood investigations (Table 1) showed mild anemia with hemoglobin of 11.2 g/dL and white cell count of 4.5 x109/L. Subsequent CD4 count of 4 cells/µL and an HIV viral load of 398,000 copies/mL, confirming AIDS. A presumptive diagnosis of Toxoplasma gondii encephalitis was made following Magnetic Resonance Imaging (MRI) of the brain (Figures 1, 2) revealed T2W/FLAIR hyperintense signals in the right thalamus (with T1 hyperintense rim enhancement), extending into the right cerebral peduncle, and the right centrum semiovale with perilesional edema. In addition, serology was positive for Toxoplasma gondii IgG. A lumbar puncture was performed to rule out other mimics like meningitis or CNS lymphoma. Opening pressure was not raised, mildly elevated protein (0.59g/L), and a normal glucose ratio. CSF cytology showed no pleocytosis and was negative for malignancy on cytology. Crucially, CSF PCR tests for tuberculosis, viruses, and bacteria were negative. However, CSF testing for Toxoplasma PCR and EBV PCR was not available in our centre.

**Table 1:** Blood and cerebrospinal fluid Investigations showing profound immunosuppression with isolated protein elevation in CSF and negative microbiologic tests. The neurological presentation, alongside a positive serum toxoplasma IgG, elevated CSF protein, and suggestive MRI findings, led to a presumptive diagnosis of cerebral toxoplasmosis. HIV: Human Immunodeficiency Virus; PCR: Polymerase Chain Reaction; IgG: Immunoglobulin G; MTB: Mycobacterium tuberculosis * = Abnormal value

Investigations	Results	Normal Range
Blood		
White cell count	4.5 x10^9^/L	4.5-11.0 x10^9^/L
CD4 count	4* cell/uL	500 -1500 cell/uL
HIV Viral load PCR	398,000* copies/mL	Not detected copies/mL
Toxoplasma IgG	Reactive*	Non-reactive
Cryptococcal Antigen	Non-reactive	Non-reactive
Rapid Plasma Reagin (RPR)	Non-reactive	Non-reactive
Cerebrospinal Fluid (CSF)		
Opening Pressure	5 cmH_2_0	10 – 20 cmH_2_O
White blood cells	No cell seen	0 – 5 cell/µL
Protein	0.59* g/L	0.15 – 0.45 g/L
Glucose	4.1 mmol/L	2.8 – 4.2 mmol/L
Meningitis panel PCR for virus & bacteria	Not detected	Not detected
MTB PCR	Not detected	Not detected
MTB Culture	No growth	No growth

Due to the unavailability of sulfadiazine, anti-toxoplasma therapy was initiated with trimethoprim-sulfamethoxazole (TMP-SMX) at a dose of 1920 mg twice daily (approximately equivalent to 10 mg/kg/day of TMP). For the movement disorder, tetrabenazine (12.5 mg twice daily) and clonazepam (1 mg twice daily) were prescribed. During the second week of anti-toxoplasma therapy, TMP-SMX was switched to pyrimethamine (75 mg daily) and clindamycin (600 mg every 6 hours) due to kidney impairment and transaminitis. On day 11 of anti-toxoplasma therapy, the patient was started on ART consisting of tenofovir disoproxil fumarate (300 mg daily), emtricitabine (200 mg daily), and dolutegravir (50 mg daily). The patient demonstrated remarkable clinical recovery prior to discharge. After one month of therapy, his involuntary movements had resolved completely, and he had regained the ability to walk independently. Following completion of the 8-week induction phase for toxoplasma therapy, maintenance therapy was initiated with clindamycin (600 mg every 8 hours) and pyrimethamine (75 mg daily).

## Discussion

*Toxoplasma gondii* is an intracellular protozoan that is typically acquired via the ingestion of tissue cysts from either uncooked meat or soil contaminated by cat faeces. Infection is often asymptomatic in immunocompetent hosts. The risk of cerebral toxoplasmosis is higher in individuals who are seropositive for the parasite than in those who are seronegative. The reactivation of latent toxoplasmosis depends on the degree of host immunosuppression. The clinical presentation varies depending on the location of brain involvement [2]. Common manifestations of Toxoplasma gondii encephalitis include headache, cranial nerve palsy, and altered sensorium, fever, focal motor deficits and seizures.

According to a systematic review on movement disorders in PLHIV, opportunistic cerebral toxoplasmosis is the most common cause of hemichorea-hemiballismus in patients with AIDS, accounting for up to 60% of hemichorea cases [6]. Cerebral toxoplasmosis frequently affects the juxta-cortical regions and basal ganglia. This specific neuroanatomical localisation is why movement disorders are a more prominent feature in toxoplasmosis than in other opportunistic infections [7]. On imaging, it typically presents as ring-enhancing lesions on contrast-enhanced CT or MRI of the brain. It can be challenging to distinguish from other conditions like cerebral tuberculoma or lymphoma radiologically [8].

The definitive diagnosis of cerebral toxoplasmosis requires a brain tissue biopsy to identify the trophozoite. However, this invasive procedure carries a high risk of bleeding and neurological complications and is therefore rarely performed. CSF Toxoplasma PCR is another diagnostic tool with good sensitivity of 83% and specificity up to 95%, but lumbar puncture is limited due to cerebral oedema, and the test is not widely available in resource-limited settings [9]. In this case, a presumptive diagnosis of Toxoplasma gondii encephalitis was made based on the clinical history of advanced HIV, the presence of a basal ganglia lesion on brain imaging, and supportive positive Toxoplasma serology, even though the classic ring-enhancing lesion was not seen.

The recommended therapy for Toxoplasma gondii encephalitis is a combination of pyrimethamine, sulfadiazine, and leucovorin [1]. However, due to the unavailability of sulfadiazine in our country, initial therapy was commenced with trimethoprim/sulfamethoxazole (TMP-SMX), which serves as an effective alternative. The regimen was then switched to pyrimethamine and clindamycin, alongside leucovorin, due to TMP-SMX-related kidney and liver impairment. Tetrabenazine and clonazepam were initiated to manage the disabling HCHB symptomatically. Tetrabenazine works by depleting monoamines in the central nervous system, helping to control hyperkinetic movements, while clonazepam acts as a muscle relaxant. Our patient demonstrated significant clinical improvement within a few weeks of therapy. This positive response further supported the presumptive diagnosis of cerebral toxoplasmosis. A good clinical outcome can be expected with an early diagnosis and prompt initiation of anti-toxoplasma therapy. 

Table 2 illustrates a summary of HCHB cases associated with cerebral toxoplasmosis and their clinical outcomes. The most commonly affected sites were the thalamus and basal ganglia. Some cases involved co-infections, particularly with tuberculosis or syphilis, which required dual therapy. The time to complete symptom resolution was highly variable, ranging from two weeks to nine months. The presence of a dual pathology often contributes to a longer recovery period than a mono-infection. In our case, the patient's symptoms resolved with treatment, which indicates that HCHB due to an active infection can be reversible [10]. This contrasts with movement disorders due to other causes, such as HIV-associated neurodegeneration or Huntington's disease (HD), which may have progressive or persistent symptoms. Therefore, a lack of clinical response to toxoplasma treatment within two weeks of therapy should raise suspicion for alternative or additional diagnoses, such as persistent gliosis [11] or an undiagnosed neurodegenerative condition like Huntington's disease.

**Table 2:** Summary of toxoplasmosis cases with HCHB, the affected sites, treatment regimens and their clinical outcomes. MMSE = Mini-Mental State Examination; HCHB : hemichorea-hemiballismus

Case, year	Age, gender	Movement disorder	Causative agent (s)	Affected central nervous system site (s)	Treatment	Time interval to Complete Resolution of Symptoms
Samira Rabhi et al. (2011) [4]	59, female	HCHB	Cerebral toxoplasmosis	Right capsule thalamic focal lesion	Trimethoprim-sulfamethoxazole, steroid	2 weeks
Reyes et al. (2016) [10]	22, male	Unilateral choreoathetosis, cognitive decline (MMSE 5) on presentation	Cerebral toxoplasmosis, secondary syphilis	Right parietal-temporal lobes, bilateral frontal, basal ganglia, thalamus region	Trimethoprim-sulfamethoxazole, intravenous penicillin for 10 days, antiretroviral therapy, carbamazepine for 6 days, phenytoin	4 months
Magano et al. (2016) [12]	26, male	Hemiballismus	Cerebral toxoplasmosis	Lower left and right cerebellar hemisphere, thalamus- mesencephalic, extension to internal capsule	Pyrimethamine, clindamycin, antituberculosis (isoniazid, rifampicin, ethambutol, pyrazinamide) surgery, stereotactic right pallidotomy on day 46, therapy due to refractory movement. An antiepileptic drug was not illustrated.	12 weeks
Dimal et al. (2021) [3]	24, male	HCHB	Cerebral toxoplasmosis, cerebral tuberculosis	Right subthalamic nucleus, cerebral peduncle	Trimethoprim/ sulfamethoxazole, anti tuberculosis (isoniazid, rifampicin, ethambutol, pyrazinamide), risperidone	9 months
Our case	36, male	HCHB	Cerebral toxoplasmosis	Right thalamus, right centrum semiovale, right cerebral peduncle	Clindamycin, pyrimethamine, folinic acid, tetrabenazine, clonazepam	1 month

## Conclusions

HCHB is one of the rare but reversible symptoms in cerebral toxoplasmosis, owing to the predilection of the infection for the basal ganglia and thalamic nuclei. The diagnosis is frequently presumptive, relying on neurological presentation, imaging, and positive toxoplasma serology. Therefore, early initiation of therapy is crucial to optimise patient outcomes. The failure of symptoms to improve with anti-toxoplasma therapy may warrant further investigation, including stereotactic brain biopsy or CSF toxoplasma PCR, to establish a definitive diagnosis. This case also highlights the importance of ART adherence to maintain virological suppression and potentially reduce the development of opportunistic infections. Medications to control the movement disorders may be beneficial as symptomatic treatment, particularly in the acute phase of the disease. The recovery time varies from weeks to months, depending on the severity of the disease and the affected sites.

## References

[1] Kaplan JE, Benson C, Holmes KK, Brooks JT, Pau A, Masur H ( 2009). Guidelines for prevention and treatment of opportunistic infections in HIV-infected adults and adolescents: recommendations from CDC, the National Institutes of Health, and the HIV Medicine Association of the Infectious Diseases Society of America. MMWR Recomm Rep.

[2] Cardoso F (2002). HIV-related movement disorders: Epidemiology, pathogenesis and management. CNS Drugs.

[3] Dimal NPM, Santos NJC, Reyes NGD, Astejada MN, Jamora RDG (2021;11:2). Hemichorea-hemiballismus as a presentation of cerebritis from intracranial toxoplasmosis and tuberculosis. Tremor Other Hyperkinet Mov (N Y).

[4] Rabhi S, Amrani K, Maaroufi M (2011). Hemichorea-hemiballismus as an initial manifestation in a Moroccan patient with acquired immunodeficiency syndrome and toxoplasma infection: A case report and review of the literature. Pan Afr Med J.

[5] Tse W, Cersosimo MG, Gracies JM, Morgello S, Olanow CW, Koller W (2004). Movement disorders and AIDS: A review. Parkinsonism Relat Disord.

[6] Amod F, Holla VV, Ojha R, Pandey S, Yadav R, Pal PK (2023). A review of movement disorders in persons living with HIV. Parkinsonism Relat Disord.

[7] Mattos JP, Rosso AL, Correa RB, Novis SA (2002). Movement disorders in 28 HIV-infected patients. Arq Neuropsiquiatr.

[8] Bowen LN, Smith B, Reich D, Quezado M, Nath A (2016). HIV-associated opportunistic CNS infections: Pathophysiology, diagnosis and treatment. Nat Rev Neurol.

[9] Alfonso Y, Fraga J, Fonseca C (2009). Molecular diagnosis of Toxoplasma gondii infection in cerebrospinal fluid from AIDS patients. Cerebrospinal Fluid Res.

[10] Reyes AJ, Ramcharan K, Aboh S, Duke N (2016). Reversible movement disorders due to toxoplasmosis as initial manifestation of HIV-AIDS, with sequential MR and video imaging. BMJ Case Rep.

[11] Achenbach J, Faissner S, Saft C (2021). Differential diagnosis of chorea-HIV infection delays diagnosis of huntington's disease by years. Brain Sci.

[12] Magano R, Jorge R, Prata M, Ventura MC, Saraiva da Cunha JG (2016). Hemiballistic movements in a newly HIV patient. IDCases.

